# METTL14-mediated m6A mRNA modification of G6PD promotes lung adenocarcinoma

**DOI:** 10.1038/s41420-024-02133-w

**Published:** 2024-08-13

**Authors:** Weidong Wu, Mengling Li, Yingxiao Wu, Qiongying Wei, Nanding Yu

**Affiliations:** 1 Department of Thoracic Surgery, Fujian Medical University Union Hospital, Fuzhou, 350001 Fujian China; 2 Fujian Key Laboratory of Cardio-Thoracic Surgery, Fujian Medical University, Fuzhou, 350122 Fujian China; 3 National Key Clinical Specialty of Thoracic Surgery, Fuzhou, 350001 Fujian China; 4 Clinical Research Center for Thoracic Tumors of Fujian Province, Fuzhou, 350001 Fujian China; 5 Department of Pulmonary and Critical Care Medicine, Fujian Medical University Union Hospital, Fuzhou, 350001 Fujian China; 6 Department of Geriatric Medicine, Fujian Medical University Union Hospital, Fuzhou, 350001 Fujian China

**Keywords:** Non-small-cell lung cancer, Non-small-cell lung cancer

## Abstract

METTL14 functions as an RNA methyltransferase involved in m6A modification, influencing mRNA biogenesis, decay, and translation processes. However, the specific mechanism by which METTL14 regulates glucose-6-phosphate dehydrogenase (G6PD) to promote the progression of lung adenocarcinoma (LUAD) is not well understood. Quantitative measurement and immunohistochemistry (IHC) analysis have demonstrated higher levels of m6A in LUAD tissues compared to adjacent normal tissues. Additionally, the expression of METTL14 was significantly increased in LUAD tissues. In LUAD cell lines, both METTL14 and m6A levels were elevated compared to normal human lung epithelial cells. Knockdown of METTL14 markedly reduced LUAD cell proliferation, migration, and invasion. Conversely, overexpression of METTL14, but not the mutant form, significantly enhanced these cellular processes in LUAD. In vivo studies using nude mice with subcutaneously transplanted LUAD cells demonstrated that stable METTL14 knockdown led to notably reduced tumor volume and weight, along with fewer Ki67-positive cells and lung metastatic sites. Importantly, METTL14 knockdown reduced glycolytic activity in LUAD cells. Through a combination of RNA sequencing and MeRIP-sequencing, we identified numerous altered genes and confirmed that IGF2BP2 enhances G6PD mRNA stability after METTL14-mediated m6A modification, thereby promoting tumor growth and metastasis. Moreover, LUAD patients with higher levels of G6PD had poorer overall survival (OS). In conclusion, our study indicates that METTL14 upregulates G6PD expression post-transcriptionally through an m6A-IGF2BP2-dependent mechanism, thereby stabilizing G6PD mRNA. These findings propose potential diagnostic biomarkers and effective targets for anti-metabolism therapy in LUAD.

## Introduction

Lung cancer (LC) is a prevalent and deadly oncological disease worldwide, characterized by high morbidity and mortality rates. It is estimated that there are approximately 2.2 million new cases and 1.8 million deaths from LC each year, making it the primary cause of cancer-related fatalities globally [1–3]. LC can be categorized into two main types: small cell lung cancer (SCLC) and non-small cell lung cancer (NSCLC). NSCLC, which constitutes about 85% of cases, includes subtypes such as adenocarcinoma, large cell carcinoma, and squamous cell carcinoma [1, 2, 4]. Among them, lung adenocarcinoma (LUAD) is the most common type of NSCLC with poor treatment response and low survival rate accompanied by rapidly pathological progresses and distal metastasis [4–7]. Therefore, comprehensive understanding the molecular mechanisms participated in the progression of LUAD is necessary for providing better diagnostic and therapeutic treatments.

N6-methyladenosine (m6A) RNA methylation, first identified in the 1970s, is a reversible modification constituting approximately half of all methylated ribonucleotides in cellular RNA [8, 9]. The m6A modification affects diverse bioprocesses such as tissue development, stemness maintenance and differentiation, DNA damage response. Up to now, a series groups of proteins are identified and verified for function of adding (writers), recognizing (readers), and removing (erasers) the methyl group to and from the RNA molecules respectively [10, 11]. If any of the processes above is out of control, it can cause abnormalities in targeted gene expression to initiate and progress of diseases such as lung, pancreas, glioblastoma, and breast cancer [9, 12–15]. So, the m6A modification can be considered as potentially and valuably diagnostic biomarkers and therapeutic targets in tumorigenesis, invasion, metastasis, and drug resistance. The m6A methyltransferase complex, consisting of core components METTL3, METTL14, and Wilms’ tumor 1-associating protein (WTAP), along with additional regulators such as RBM15, KIAA1429, and potentially ZC3H13 and CBLL1, functions as the writer responsible for catalyzing m6A modification in mammals [16, 17]. METTL3 was elucidated that has multiple and critical function in promoting cell proliferation, migration, and invasion through the regulating expression of oncogenes and tumor suppressor genes [18–20]. METTL14, as an essential allosteric activator of METTL3, also plays a significant role in tumor progression across various cancer types [21–23]. In contrast, the demethylases, including fat mas and obesity-associated (FTO) and AlkB homolog 5 (ALKBH5) functioning as ‘erasers’ to remove m6A from mRNA [17, 24, 25]. FTO was shown to regulate MZF1 expression by decreasing m6A levels and mRNA stability, thereby promoting tumor progression in lung squamous cell carcinoma [26]. Additionally, members of the YT521-B homology (YTH) domain family proteins (YTHDFs and YTHDCs), insulin-like growth factor 2 mRNA-binding proteins (IGF2BPs), and eukaryotic initiation factor 3 (eIF3) act as ‘readers’ of m6A modifications, promoting mRNA stability and translation [27, 28]. For instance, YTHDF2 interacts with the 3’-untranslated region (UTR) of 6-phosphogluconate dehydrogenase (6PGD) to enhance mRNA translation, thereby promoting lung cancer cell proliferation [29]. However, comprehensive data on the expression, regulation, and clinical relevance of m6A-related genes in LUAD have been insufficient. Recent studies indicate that m6A and its associated proteins are pivotal in tumorigenesis and cancer progression across various cancer types, including lung cancer [17]. For example, METTL3 is crucial for TGF-β-induced epithelial-mesenchymal transition in lung cancer cells, whereas YTHDF2 promotes lung cancer cell growth by enhancing the translation of 6PGD mRNA [30]. However, the biological significance and underlying mechanism of m6A in LUAD remain poorly understood.

In this study, we found the content of m6A in LUAD patient tissues and LUAD cells collaborate with significantly elevated expression of METTL14. Knockdown METTL14 remarkably decreased cell proliferation, invasion, and migration in vitro and in vivo. Furthermore, when mutation METTL14 at position 298 (METTL14-R298P) for methyltransferase complex recognition, substantially abolished its functions. In contrast, METTL14 overexpression obviously promoted LUAD cell growth and metastasis. Mechanistically, METTL14 post-transcriptionally up-regulates G6PD expression for LUAD glycolytic metabolism via an m6A-IGF2BP2-dependent manner to further promote stabilization of G6PD mRNA. Taken together, our research reveals critical function and mechanism of METTL14 in LUAD metastasis, provides a potential diagnostic biomarkers and effective targets for LUAD therapy.

## Results

### Elevated expression of METTL14 accompanying with high levels of m6A in LUAD patients and LUAD cells

**Fig. 1:**
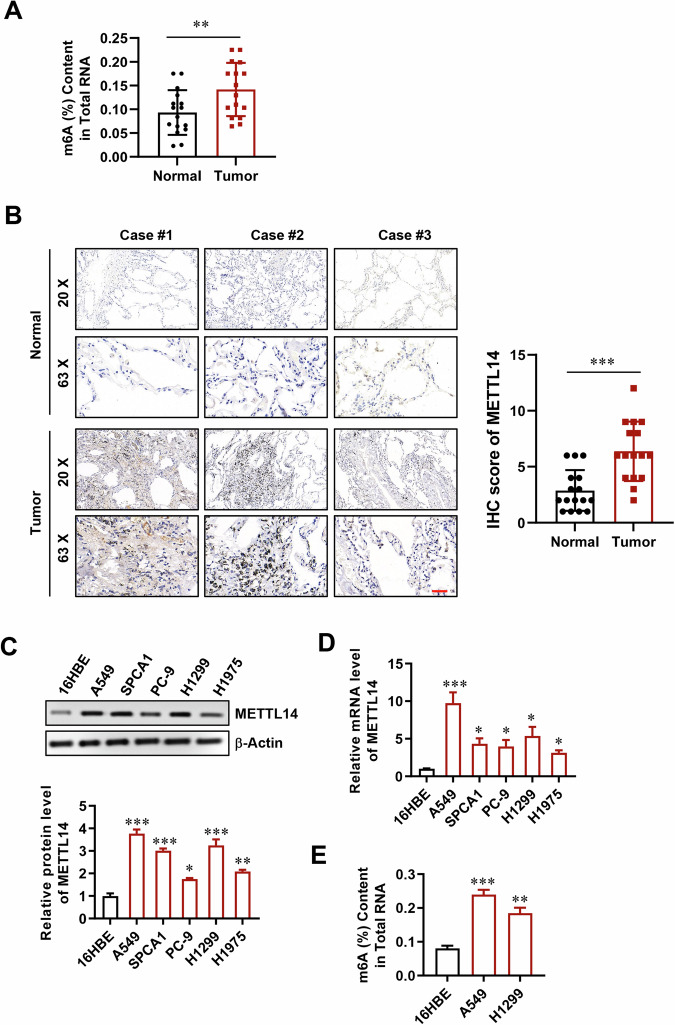
Methyltransferase-like 14 (METTL14) modulates N6-methyladenosine (m6A) levels in both lung adenocarcinoma (LUAD) tissues and LUAD cells. **A** Quantitative assessment of m6A content in LUAD and adjacent normal tissues (*n* = 16). **B** Immunohistochemistry (IHC) analysis showing METTL14 expression in LUAD and adjacent normal tissues (*n* = 16). Scale bar, 100 μm. **C** Western blot (WB) analysis of METTL14 protein levels in various lung cancer cells (*n* = 3). **D** qPCR analysis of METTL14 expression in various lung cancer cells (*n* = 3). **E** Quantitative measurement of m6A content in LUAD cells and normal human lung epithelial cells (*n* = 3). *p < 0.05, **p < 0.01, ***p < 0.001.

Previous studies have reported dysfunction of m6A modification in LUAD tissues [31–33]. Therefore, we quantitatively assessed the m6A content in samples obtained from LUAD tissues, revealing a significant increase compared to adjacent normal tissues (Fig. 1A). Immunohistochemistry (IHC) analysis further validated these findings (Fig. S1A). Additionally, the expression of METTL14, a key methyltransferase involved in m6A modification, was notably higher in LUAD tissues than in adjacent normal tissues (Fig. 1B). In LUAD cell lines, RT-qPCR and Western blot analysis demonstrated elevated mRNA and protein levels of METTL14 compared to normal human lung epithelial cells 16HBE (Fig. 1C, D). Furthermore, we selected two LUAD cell lines exhibiting the highest METTL14 expression to quantify m6A content, which was found to be elevated relative to 16HBE cells (Fig. 1E). Collectively, these findings indicate that METTL14 likely regulates m6A levels in both LUAD patients and LUAD cell lines.

### METTL14 promotes cell proliferation and migration in LUAD cells

**Fig. 2:**
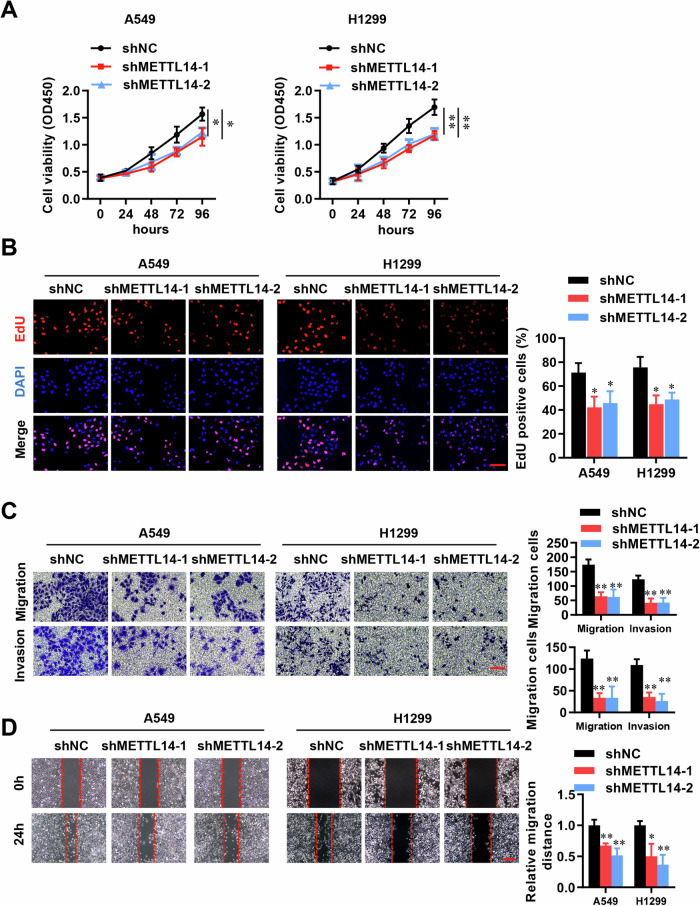
Knockdown of METTL14 inhibits cell proliferation and migration in LUAD cells. **A** Cell viability analysis following METTL14 knockdown in LUAD cells (*n* = 3). **B** Immunofluorescence (IF) analysis of cell proliferation in METTL14 knockdown LUAD cells (*n* = 5). Scale bar, 100 μm. **C** Analysis of invasion in METTL14 knockdown LUAD cells (*n* = 5). Scale bar, 100 μm. **D** Analysis of migration in METTL14 knockdown LUAD cells (*n* = 5). Scale bar, 100 μm. *p < 0.05, **p < 0.01.

**Fig. 3:**
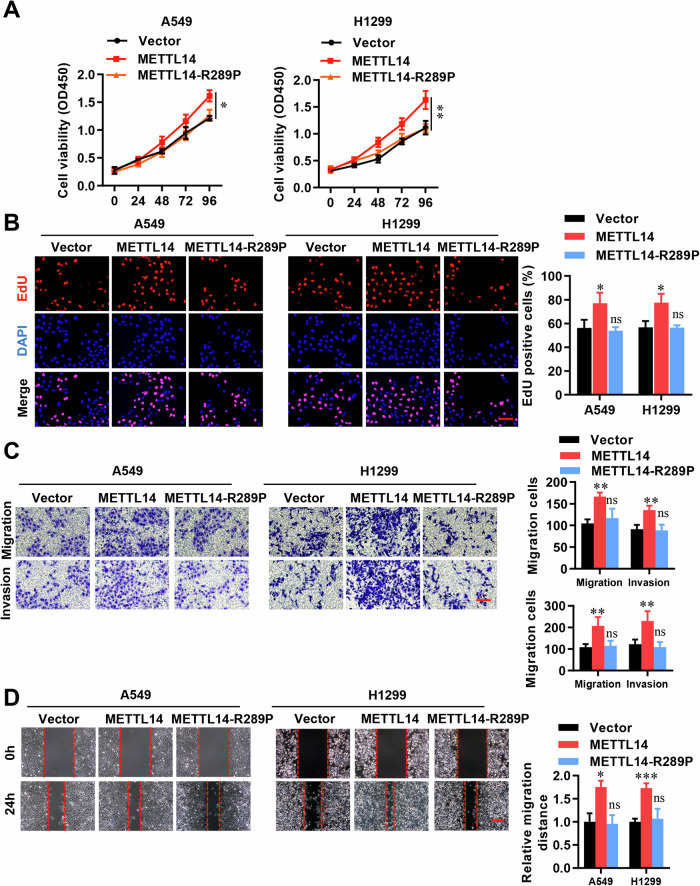
METTL14 regulates cell proliferation and migration in LUAD cells via m6A modifications. **A** Cell viability analysis after METTL14 overexpression or mutant R289P in LUAD cells (*n* = 3). **B** IF analysis of cell proliferation in LUAD cells with METTL14 overexpression or mutant R289P (*n* = 5). Scale bar, 100 μm. **C** Analysis of invasion in LUAD cells with METTL14 overexpression or mutant R289P (*n* = 5). Scale bar, 100 μm. **D** Analysis of migration in LUAD cells with METTL14 overexpression or mutant R289P (*n* = 5). Scale bar, 100 μm. ns, not significant, *p < 0.05, **p < 0.01, ***p < 0.001.

To investigate the impact of METTL14 on the malignancy of LUAD, we employed shRNAs to knockdown METTL14 in A549 and H1299 cell lines (Fig. S2). This resulted in a significant reduction in cell viability, as determined by CCK-8 assays (Fig. 2A). Further analysis using EDU staining confirmed that METTL14 knockdown led to decreased cell proliferation (Fig. 2B). Cell invasion was assessed using transwell assays, while migration was evaluated using wound healing assays. Compared to the shNC group, both shMETTL14-1 and shMETTL14-2 notably inhibited cell migration and invasion (Fig. 2C, D). Conversely, overexpression of METTL14 significantly promoted cell proliferation (Fig. 3A, B) and invasion (Fig. 3C, D). Furthermore, when we mutated METTL14 at position 298 (METTL14-R298P), which is critical for target recognition of the methyltransferase complex [34], substantially abolished its functions in vitro (Fig. 3A–D). These findings suggest that the impact of METTL14 on LUAD cell proliferation and migrative processes is dependent on its function as an RNA methylation modifier.

### METTL14 deficiency decelerates LUAD cell growth and metastasis

**Fig. 4:**
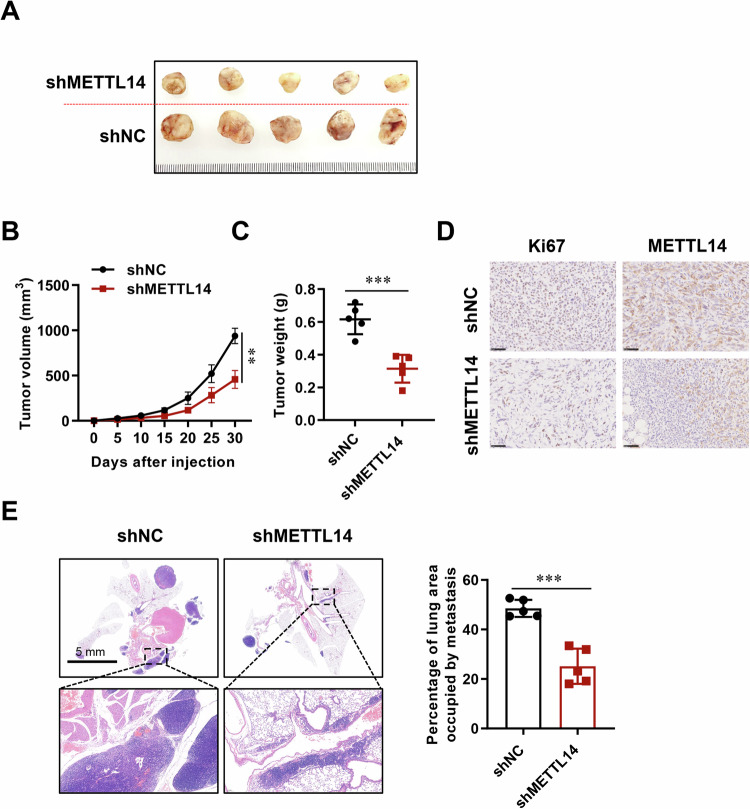
METTL14 deficiency attenuates tumor growth and metastasis in vivo. **A**–**C** Morphological and size analysis of xenografted tumors following subcutaneous injection of METTL14 knockdown LUAD cells (*n* = 5). **D** IHC analysis of cell proliferation in xenografted tumor tissues (*n* = 5). Scale bar, 5 mm. **E** Hematoxylin and eosin (HE) staining and analysis of tumor metastasis in lung tissues after subcutaneous injection of METTL14 knockdown LUAD cells (*n* = 5). Scale bar, 100 μm. ns, not significant, *p < 0.05, **p < 0.01, ***p < 0.001.

To assess the consistency of our results following METTL14 knockdown in an in vivo setting, we subcutaneously transplanted A549 cells with stable METTL14 knockdown into nude mice. After 30 days, the depletion of METTL14 significantly restricted tumor volume and weight compared to the shNC group (Fig. 4A–C). IHC analysis further confirmed that METTL14 knockdown led to a lower number of Ki67 positive cells in tumor tissue sections (Fig. 4D). Additionally, HE staining demonstrated that inhibiting METTL14 reduced the number of lung metastatic sites following cell injection (Fig. 4E). These findings collectively indicate that elevated levels of METTL14 contribute to accelerated cancer cell proliferation in LUAD.

**Fig. 5:**
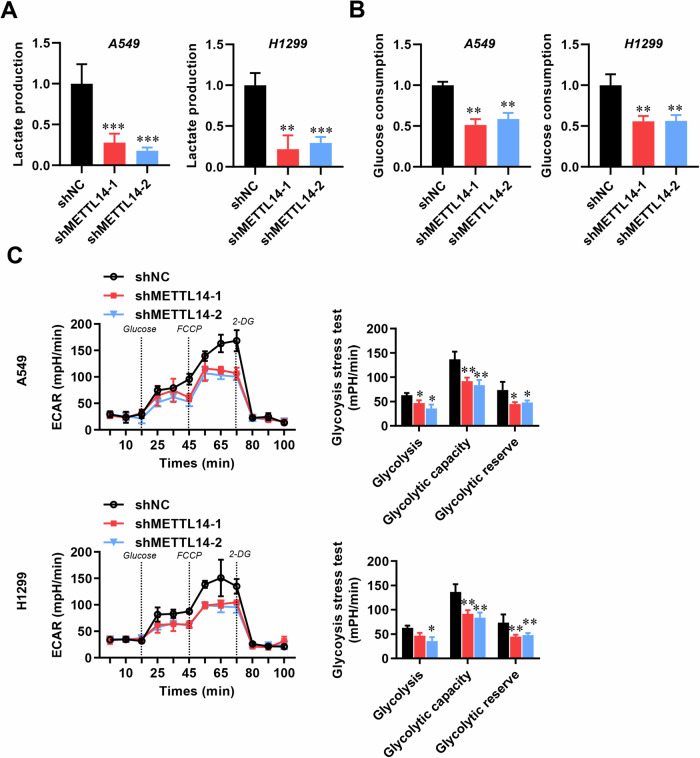
METTL14 influences glycolysis in LUAD cells. **A** Lactate production in LUAD cells with or without METTL14 knockdown (*n* = 5). **B** Glucose consumption in LUAD cells with or without METTL14 knockdown (*n* = 5). **C** Extracellular acidification rate (ECAR) measured in LUAD cells with or without METTL14 knockdown (*n* = 5). *p < 0.05, **p < 0.01, ***p < 0.001.

Numerous studies have revealed that the aerobic glycolytic phenotype in LUAD is highly correlated with tumor formation, progression, and metastasis [20, 35]. Thus, we sought to investigate whether METTL14 regulates the glycolytic process in LUAD. Encouragingly, we observed that METTL14 knockdown resulted in reduced lactate production and glucose uptake in both A549 and H1299 cells compared to the control group (Fig. 5A, B). Furthermore, the kinetic profiles of ECAR demonstrated a significant decrease in glycolytic function upon METTL14 loss, accompanied by lower glycolysis, glycolytic capacity, and glycolytic reserve (Fig. 5C).

### G6PD is a target of METTL14 mediated m6A modification

**Fig. 6:**
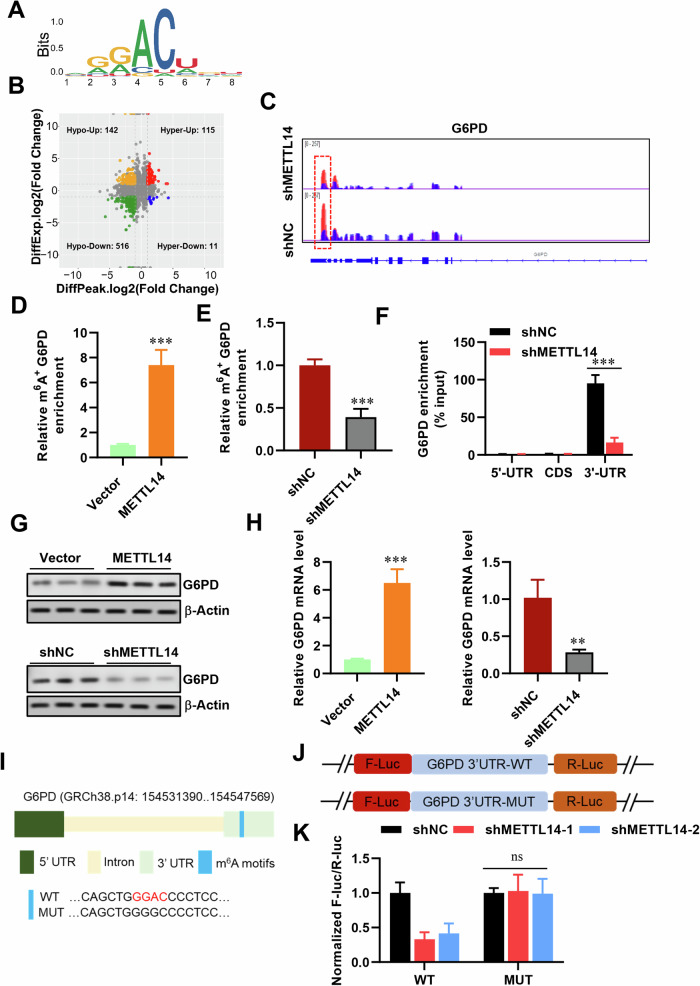
G6PD is targeted by METTL14-mediated m6A modification. **A** Consensus motif identified in LUAD cells by MeRIP-seq. **B** Volcano plot showing DEGs identified by RNA-seq in LUAD cells with or without METTL14 knockdown. **C**–**E** Relative abundance of m6A peaks in G6PD mRNA analyzed by MeRIP-seq in LUAD cells. **F** Enrichment binding sites of m6A signals in G6PD mRNA. **G** Western blot and analysis of G6PD protein levels in LUAD cells (*n* = 3). **H** RT-qPCR analysis of G6PD expression in LUAD cells (*n* = 3). **I** Sequence motifs in m6A-modified sites of G6PD mutants. **J**, **K** Sequence motifs and cellular luciferase assay in G6PD cells or 3’-UTR mutant LUAD cells with or without METTL14 knockdown (*n* = 3). ns, not significant, *p < 0.05, **p < 0.01, ***p < 0.001.

**Fig. 7:**
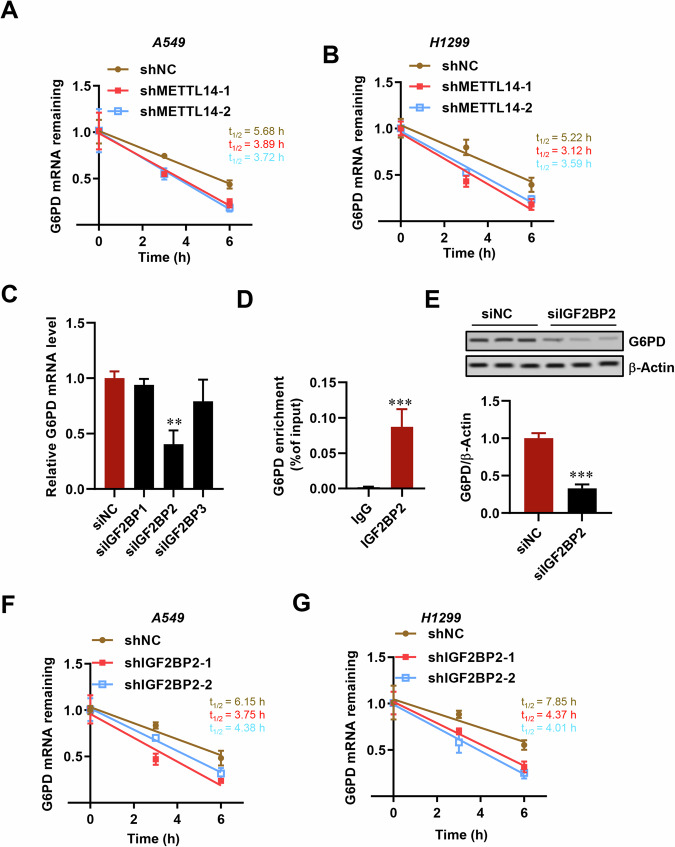
IGF2BP2 enhances G6PD mRNA stability. **A**, **B** Half-life assay of G6PD mRNA in LUAD cells with or without METTL14 knockdown (*n* = 3). **C** RT-qPCR analysis of G6PD expression in LUAD cells with or without IGF2BPs knockdown (*n* = 3). **D** RIP-qPCR validation of IGF2BP2 binding to G6PD mRNA. **E** Western blot analysis of G6PD expression after IGF2BP2 knockdown (*n* = 3). **F**, **G** Half-life assay of G6PD mRNA in LUAD cells with IGF2BP2 knockdown (*n* = 3). *p < 0.05, **p < 0.01, ***p < 0.001.

To identify specific glycolytic targets under that high METTL14 accelerates tumor growth and metastasis in LUAD, combining RNA-sequencing with MeRIP-sequencing was employed by using stable METTL14-knockdown and NC vector transfected LUAD cells. We recognized the consensus motif which which was highly concentrated in m6A sites (Fig. 6A) and identified 516 altered genes which both hypo-m6A-peaks and decreased expression upon METTL14 knockdown (Fig. 6B). Among these 516 genes, we specifically focused on the glycolysis-related gene G6PD. Our previous research has revealed a significant correlation between G6PD and the poor prognosis of lung adenocarcinoma patients. Meanwhile, we verified the METTL14-binding peaks near the G6PD regions (Fig. 6C). Previous researches proved that G6PD can be methylated and promoted translation by m6A ‘readers’ at 3’-UTR to promotes lung cancer cell growth [29], therefore we detected m6A level in G6PD (Fig. S1B) and found that it significantly up-regulated after METTL14 overexpression (Fig. 6D), whereas knockdown METTL14 reversed this phenotype (Fig. 6E) in consistent with lower binding at 3’-UTR of G6PD (Fig. 6F). Therefore, manipulating METTL14 significantly alters G6PD expression at both the mRNA and protein levels (Fig. 6G, H). Introducing mutations to the m6A motifs at G6PD’s 3’-UTR (Fig. 6I) abolished the binding efficiency between METTL14 and G6PD (Fig. 6J, K). Furthermore, the mRNA half-life of G6PD was reduced in tumor cells with METTL14 downregulation (Fig. 7A, B). These findings strongly support that METTL14 promotes tumor growth and metastasis through m6A modification of G6PD.

### IGF2BP2 enhances G6PD mRNA stability via an m6A-dependent manner

Previous studies have suggested that IGF2BP family proteins function as m6A ‘readers’ that enhance mRNA stability [27, 28]. To further elucidate the complete modification mechanism of METTL14-G6PD in LUAD, we silenced IGF2BP1, 2, and 3 in tumor cells using siRNA transfection. The knockdown efficiency was confirmed by RT-qPCR, revealing that only siIGF2BP2 markedly reduced G6PD mRNA levels (Fig. 7C). Conversely, overexpression of IGF2BP2 significantly increased G6PD expression (Fig. 7D). Additionally, upon treating tumor cells with siIGF2BP2, we observed a decrease in G6PD protein levels (Fig. 7E). Furthermore, the mRNA half-life of G6PD was shortened in LUAD cells with IGF2BP2 downregulation (Fig. 7F, G), indicating that IGF2BP2 plays a critical role in recognizing METTL14-mediated m6A methylation on G6PD mRNA and enhancing its stability and expression.

### METTL14‑mediated m6A modification of G6PD promotes LUAD progression

**Fig. 8:**
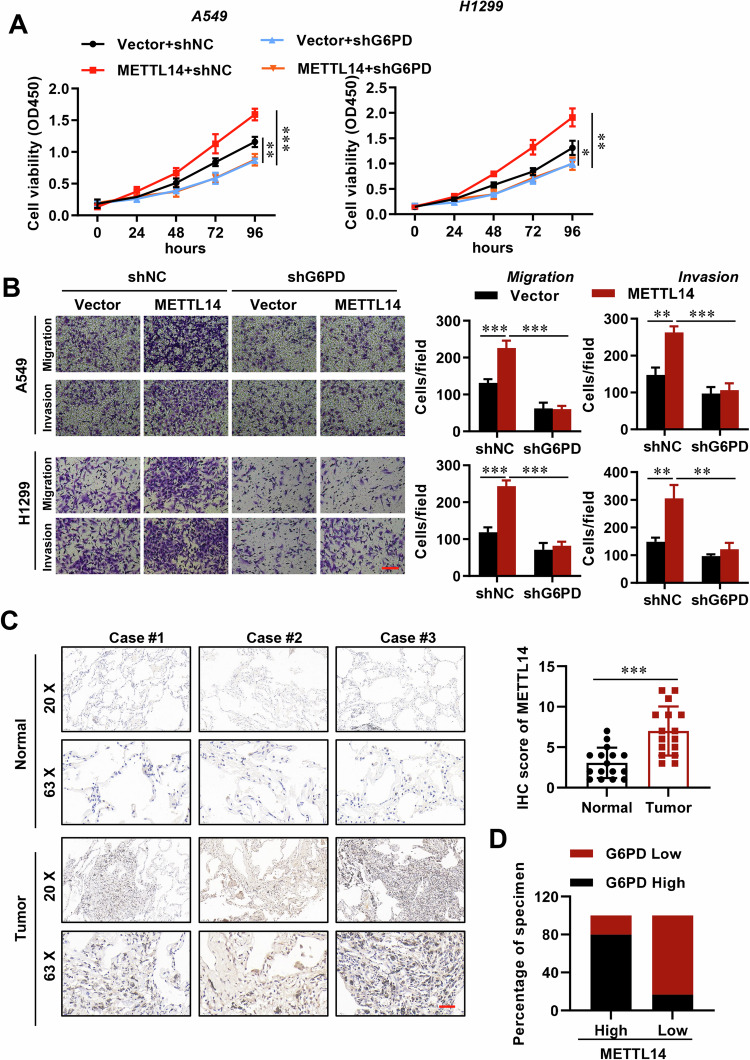
METTL14-mediated G6PD promotes LUAD progression. **A** Cell viability analysis of WT and METTL14-overexpressing LUAD cells with or without G6PD knockdown (*n* = 3). **B** Morphological analysis of WT and METTL14-overexpressing LUAD cells with or without G6PD knockdown (*n* = 5). Scale bar, 100 μm. **C** IHC analysis of G6PD expression in LUAD and adjacent normal tissues stratified by METTL14 expression (*n* = 5). **D** IHC analysis of correlation between METTL14 expression and G6PD levels in LUAD tissues. Scale bar, 100 μm. ns, not significant, *p < 0.05, **p < 0.01, ***p < 0.001.

Subsequently, we explored the impact of reducing G6PD levels in LUAD cells and observed that this effectively counteracted the enhancement of cell proliferation (Figs. 8A, S3A), migration (Fig. 8B), and invasion (Fig. S3B) induced by METTL14 overexpression. Additionally, immunohistochemical (IHC) analysis revealed a significant increase in G6PD levels in LUAD tissues (Fig. 8C). Importantly, we identified a positive correlation between METTL14 expression and G6PD levels in LUAD tissues (Fig. 8D). Furthermore, utilizing the Kaplan-Meier Plotter tool for online analysis, we determined that higher G6PD levels were associated with poorer overall survival in LUAD patients (Fig. S3C). In summary, our findings demonstrate that METTL14-IGF2BP2 promotes G6PD expression by recognizing and mediating m6A methylation of G6PD mRNA, thereby enhancing its stability in LUAD.

## Discussion

This study has identified the critical role of up-regulated METTL14 in enhancing gene expression of G6PD, thereby promoting tumor growth and metastasis in LUAD. We observed a significant increase in METTL14 expression in tumor tissues from LUAD patients compared to adjacent normal tissues, along with high levels of m6A modification. Knockdown of METTL14 effectively suppressed tumor cell proliferation, migration, and invasion both in vitro and in vivo. Conversely, overexpression of METTL14 accelerated LUAD-related phenotypes. Mechanistically, we found that METTL14 up-regulates G6PD expression through an m6A-IGF2BP2-dependent pathway, contributing to tumor growth and metastasis in glycolytic microenvironments. These findings illuminate the role and mechanism of METTL14 in promoting m6A-mediated gene activation during LUAD pathogenesis. Moreover, our results suggest that targeting G6PD, as a downstream effector of METTL14, holds promise as a meaningful therapeutic strategy for developing treatments for LUAD.

Abnormal m6A RNA modification levels are associated with tumorigenesis, proliferation, metastasis, and invasion [8, 9, 11, 36]. Here we found that high m6A both in LUAD tissues and LUAD cell lines. Furthermore, METTL14 serves as the core component of the m6A methyltransferase complex, participating in the dynamic and reversible process of m6A modification [13, 23, 33]. Ren and his colleagues found that METLL14 significantly higher expressed in the NSCLC tumor than in the normal group, and was significantly correlated with differentiation stages of NSCLC [33]. However, another study reported decreased expression of MELLT3, METTL14, and ALKBH5 mRNA in LUAD tissues compared to adjacent non-tumor tissues, with no differences observed at the protein level [31]. Noticeably, they also pointed that the limitation about small number of specimens used may affect the accuracy of their data in some degree. Recently, Zhang et al. found that METTL14 was reduced in LUAD and not affect the prognosis of LUAD patients [37]. Here we demonstrated that evaluating METTL14 promotes the m6A level both in LUAD patients and LUAD cells, which indicated its key role in LAUD pathological progression. Given the presence of certain discrepancies in the above findings, it is important to acknowledge that m6A modification is a dynamic and reversible process that can be influenced by various conditions or stages [31, 33, 38]. Therefore, further evidence and validation are required to support our findings in future studies.

METTL14 plays dual roles as both an oncogene and a tumor suppressor gene in regulating the onset and progression of various cancers [39–41]. Our data has shown that down-regulating of METTL14 efficiently impaired cancer cell growth and metastasis in vivo and in vitro. More important, these functions depend on up-regulation of m6A level in glycolytic process. Elevation glycolysis, which involves increased glucose uptake and lactate production, is regarded as energy adaptation of cancer cells to provide continuous energy for cell proliferation, invasion, and migration under hypoxic microenvironment [42, 43]. Ma and colleagues reported that upregulation of METTL3-YTHDF1 mediates the translation of enolase 1 (ENO1) through m6A-dependent modification, stimulating glycolysis and tumorigenesis in LUAD [35]. Phospholysine phosphohistidine inorganic pyrophosphate phosphatase (LHPP) has been demonstrated to inhibit the Wnt pathway and reduce glycolytic metabolism. However, in gastric cancer cells, LHPP is repressed due to m6A modification mediated by METTL14 [21]. Furthermore, it has been found that METTL14 can inhibit aerobic glycolysis in p53-wild-type colorectal cancer cells by suppressing the expression of SLC2A3 and PGAM1 in an m6A-dependent manner [22]. This suggests that METTL14 might target genes involved in glycolysis to promote cancer cell growth and metastasis in LUAD.

To elucidate the regulatory mechanism of METTL14 in the glycolytic process of LUAD, we screened potential targets and validated them at both the gene and protein levels. Interestingly, METTL14 upregulates the expression of G6PD through m6A modification. G6PD is known to play a fundamental role in metabolic reprogramming of cancer cells [44–46]. Knockdown of G6PD efficiently negated the promotion of LUAD cell proliferation, migration, and invasion caused by METTL14 overexpression. Previous research has indicated that METTL14-binding peaks near the G6PD 3’-UTR regions can undergo methylation, thereby promoting translation facilitated by m6A ‘readers’ such as YTHDF2, which in turn enhances lung cancer cell growth [29]. Besides, IGF2BP family proteins can also act as m6A ‘readers’ to enhance mRNA stability and translation [6, 27, 28]. When we knocked down IGF2BP1, 2 and 3 in tumor cells via siRNA transfection, only siIGF2BP2 markedly decreased the G6PD mRNA and protein expression level through reducing mRNA stability. Conversely, overexpressing IGF2BP2 significantly enhanced G6PD expression. Therefore, we partially elucidate the function and mechanism of METTL14 in LUAD progression by ‘writing’ m6A in G6PD gene to enhance G6PD expression accompany with IGF2BP2 ‘reading’. Certainly, the original source/reason of high METTL14 expression in LUAD is unknown. Further, there may be other METTL14 downstream targets to regulate migration and invasion in LUAD need to be explored.

## Conclusion

Our results demonstrated that METTL14 markedly elevated m6A levels on G6PD in both LUAD tissues and cells. Furthermore, IGF2BP2 was identified as crucial for stabilizing G6PD, promoting tumor growth and metastasis. Therefore, targeting the METTL14-IGF2BP2-G6PD pathway could represent a promising therapeutic strategy for treating human LUAD.

## Materials and methods

### Clinical sample collection

All specimens of LUAD tissues and paired adjacent normal lung tissues were obtained from enrolled patients who provided informed consent. Tissues were promptly frozen in liquid nitrogen post-removal for subsequent experiments, conducted with approval from the Ethics Committee of Fujian Medical University Union Hospital (Fuzhou, China).

### Cell culture

Cell lines (16HBE, A549, SPCA1, PC-9, H1299, and H1975) were procured from the Cell Bank of the Chinese Academy of Sciences (Shanghai, China). A549, H1975, and H1299 cells were cultured in RPMI-1640 medium (Thermo, USA) supplemented with 10% FBS, while 16HBE, SPC-A1, and PC9 cells were cultured in DMEM medium (Thermo, USA) supplemented with 10% FBS. All cell cultures were maintained at 37 °C in a 5% CO2 atmosphere, with media containing 100 U/mL penicillin and 100 U/mL streptomycin.

### Transfection

Lentiviral vectors containing METTL14 shRNA were obtained from General Biosystems (Anhui, China). Cells were seeded in a 6-cm dish at a density of 2.5 × 10^6 cells, allowed to adhere for 12 h, and transfected with either overexpression plasmid (2 μg) or siRNA (1.5 μg) targeting each gene using Lipofectamine® 2000 reagent (Thermo, USA) as per the manufacturer’s instructions.

### Metabolic assay and extracellular acidification rate (ECAR) analysis

Glucose and lactate concentrations in the cultured media were quantified using commercial kits (BioVision) as per the manufacturer’s protocols. The ECAR was analyzed by using the Seahorse XF24 instruments as previously reported [36]. In brief, 1×10^5 A549 and H1299 cells with or without METTL14 knockdown were cultured in Seahorse Bioscience assay medium supplemented with 2 mM glutamine for 1.5 h at 37 °C. The cells were subsequently analyzed using the Glycolytic Stress Test Kit (Seahorse Bioscience) and Seahorse XF96 Wave software.

### Luciferase reporter assay

The 3’-untranslated regions (3’-UTR) of G6PD mRNA were PCR-amplified and subsequently cloned into the pmirGLO luciferase vector (Promega). Mutant constructs were generated using the Quick-Change II Site-Directed Mutagenesis Kit (Agilent). For the luciferase reporter assay, LUAD cells were co-transfected with shMETTL14 or NC along with wild-type or mutant luciferase constructs. Luciferase activity was measured using the dual-luciferase reporter assay kit (Promega).

### Cell proliferation, migration, and invasion assay

A549 and H1299 cells were seeded in 96-well plates for various treatments. After 24 h, 20 μL of CCK8 (Beyotime) was added to each well, and the optical density (OD) at 450 nm was measured using a spectrophotometer following 1 h of incubation. Simultaneously, cell proliferation was assessed using the EdU Cell Proliferation Kit (Beyotime) according to the manufacturer’s protocol.

For cell migration analysis, a wound-healing assay was performed by creating artificial wounds in A549 and H1299 cells using a pipette tip after various treatments. The cells were then cultured in 2% FBS for 20 h, and images were captured at the beginning and after 20 h to monitor wound closure. Cell invasion was evaluated using 24-well Transwell plates as previously described [47]. After 24 h of incubation, migrated cells were fixed rapidly, stained with 0.5% crystal violet, and photographed and counted.

### Establishment xenografted model in mouse

All mice were managed in accordance with NIH guidelines for laboratory animal care (approved by the Ethics Committee of Fujian Medical University Union Hospital, Fuzhou, China). They were housed in standard cages at 22 °C with a 12-12-h light-dark cycle, under specific pathogen-free conditions with ad libitum access to food and water. Thymus-free nude mice were utilized for LUAD cell transplantation. A549 cells, suspended in DMEM medium at a concentration of 1 × 10^7 cells/mL, were subcutaneously injected into the right flank of nude mice. Tumor volumes were monitored every five days and calculated using the formula: Tumor volume = (W^2 × L)/2, where L is the longest tumor diameter and W is the shortest tumor diameter, as previously described [36].

To study LUAD cell metastasis in vivo, 10^6 A549 cells were injected intravenously into the lateral tail vein of 8-week-old BALB/c nude mice. After 30 days, lung tumor nodules were counted. Mice were euthanized, and lung tissues were collected, dissected, and stained with H&E to examine tumor colony histomorphology. Images were captured to measure the relative lung area occupied by metastases.

### RT-qPCR

Total RNA was extracted from all samples using Trizol reagent (Thermo Fisher Scientific) through phenol-chloroform precipitation. m6A levels were assessed with the EpiQuik m6A Methylation Quantification Kit (Epigentek) according to the manufacturer’s guidelines. After eliminating genomic DNA and performing reverse transcription, qRT-PCR was conducted using an ABI PRISM 7900 system (Applied Biosystems) with SYBR Green Real-Time PCR Master Mix Plus (TOYOBO). mRNA expression was normalized to GAPDH. Primer sequences for RT-qPCR are listed in Supplementary Table 1.

### Methylated RNA immunoprecipitation sequencing (MeRIP-seq) and MeRIP-qPCR

Intact mRNA was isolated from total RNA samples using the mRNA Isolation Kit (Promega) following the manufacturer’s protocol, yielding more than 5 μg of purified mRNA. MeRIP was performed using the Magna MeRIP™ m6A Kit (Millipore) as per the manufacturer’s instructions. Initially, the isolated mRNA was fragmented into 200-nucleotide-long fragments by incubating at 94 °C for 5 min, and the size of these fragments was verified using an Agilent 2100 Bioanalyzer (Agilent, CA, USA). Subsequently, m6A-methylated mRNAs were immunoprecipitated with an m6A-specific antibody, followed by standard procedures of immunoprecipitation, washing, and elution. The eluted RNA and MeRIPed RNA were subjected to deep sequencing using an Illumina Novaseq™ 6000 platform at LC-BIO Bio-techltd (Hangzhou, China), following the vendor’s protocol. Immunoprecipitated samples were also analyzed by MeRIP-qPCR.

### RNA binding protein immunoprecipitation (RIP)-qPCR

The RIP assay utilized the MagnaTM RIP RNA-Binding Protein Immunoprecipitation Kit (Millipore). Initially, cell lysates were incubated overnight at 4 °C with beads coated with 5 μg of IGF2BP2 antibodies (ab128175, Abcam) under rotation. RNA-protein-magnetic bead complexes were subsequently washed and eluted using proteinase K digestion buffer. Immunoprecipitated RNA was then extracted via phenol-chloroform RNA extraction methods. Enriched RNA was analyzed by qPCR and normalized against input levels.

### RNA stability assays

Cells were seeded overnight in 12-well plates and treated with 5 μg/mL actinomycin D (MedChemExpress) at 0, 3, and 6 h. Total RNA was isolated using TRIzol (Invitrogen), followed by qPCR analysis. mRNA expression levels for each group at every time point were calculated and normalized to GAPDH.

### Western blot (WB) and immunohistochemistry (IHC) staining

Cells were lysed by using RIPA lysis buffer (Beyotime) on ice after different treatments. Protein concentrations were measured by using BCA kit (Beytime). The membrane blots were blocked with 5% milk, followed by incubated with a primary antibody of METTL14 (ab309096, Abcam), G6PD (ab210702, Abcam), and β-Actin (ab178787, Abcam) overnight in 4 °C. After rinsing with PBST, secondary antibody goat anti-mouse or goat anti-rabbit (Jackson) were used. Protein bands were visualized using an ECL kit (Perkin Elmer) for analysis.

For IHC staining, the samples were washed with cold PBS, fixed overnight with 4% paraformaldehyde (PFA), and embedded in OCT (SAKURA) compound in −80 °C. Embedded samples were then cut into 5 µm/slices. Slices were stained with primary antibodies against METTL14 (1:500), m6A (1:500), G6PD (1:500), and ki67 (1:200) (Abcam) respectively. The staining scores of ISH/IHC were evaluated by multiplying the positive ratio with the staining intensity. The positive region was categorized into five groups: <5%, 5–25%, 26–50%, 51–75%, or >75%, assigned values of 0, 1, 2, 3, or 4, respectively. Staining intensity was determined as negative (0), weakly positive (1), moderately positive (2), or strongly positive (3). The IHC assessment was conducted independently by two experienced pathologists.

### Statistical analysis

Statistical analysis was performed using GraphPad Prism software, and data are presented as mean ± SD. Student’s *t*-test was employed to assess differences between two groups, while one-way ANOVA was utilized for comparisons involving three or more groups. A value of P < 0.05 was considered statistically significant.

### Supplementary information

supplementary figure

Supplementary Table 1

Full and uncropped western blots

## Data Availability

All necessary data to assess the conclusions are included in the paper. Additional related data can be obtained by contacting the corresponding author.
