# Supplementary material for: METTL14-mediated m6A mRNA modification of G6PD promotes lung adenocarcinoma
Source: Cell Death Discov. 2024 Aug 13;10:361. doi: 10.1038/s41420-024-02133-w (PMC11322390; doi:10.1038/s41420-024-02133-w)
Supplement: Supplementary file 1 — supplementary figure [file 41420_2024_2133_MOESM1_ESM.pptx]

## Slide 1
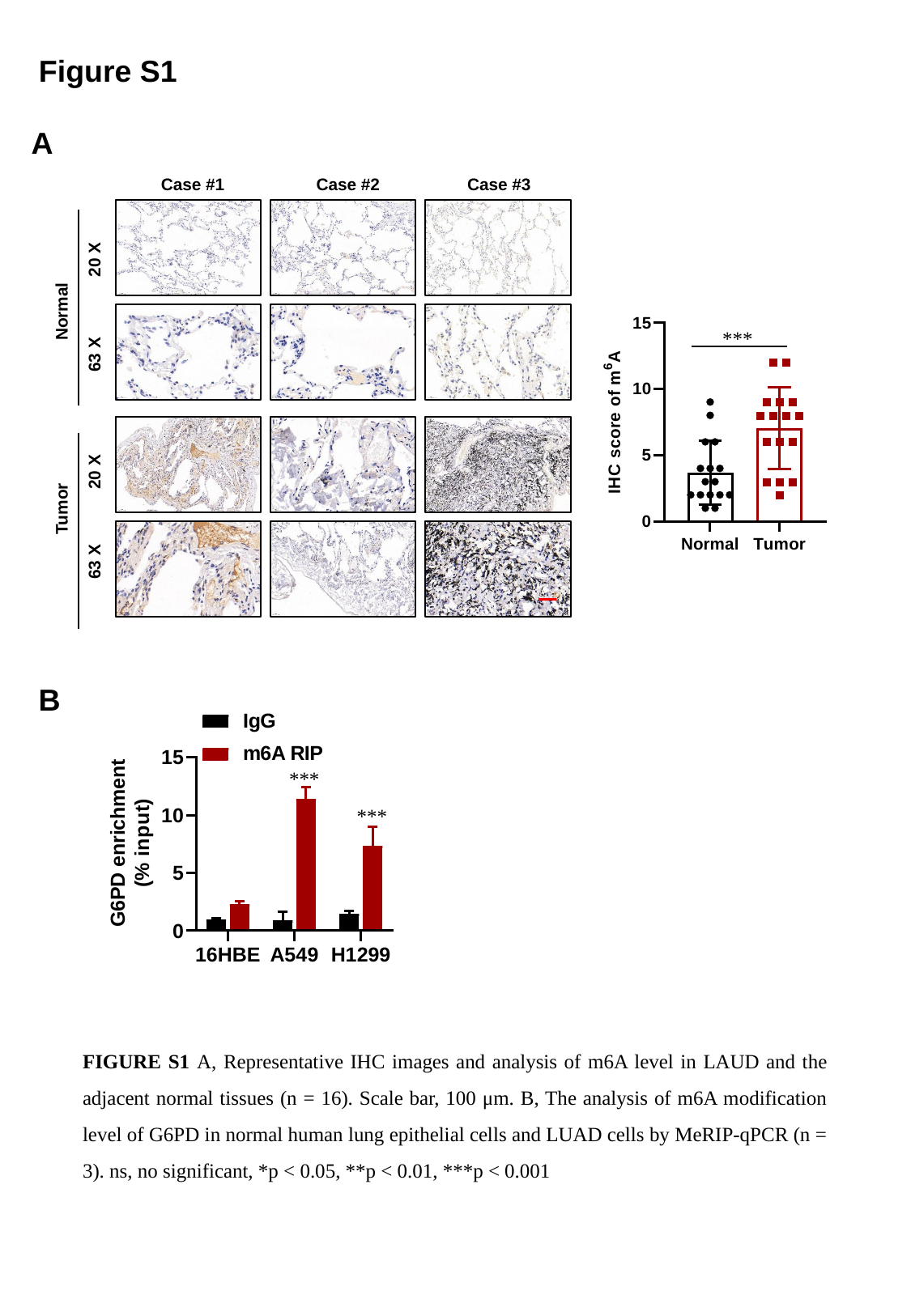

Figure S1
A
Case #1
Case #2
Case #3
20 X
Normal
***
63 X
20 X
Tumor
63 X
B
***
***
FIGURE S1 A, Representative IHC images and analysis of m6A level in LAUD and the adjacent normal tissues (n = 16). Scale bar, 100 μm. B, The analysis of m6A modification level of G6PD in normal human lung epithelial cells and LUAD cells by MeRIP-qPCR (n = 3). ns, no significant, *p < 0.05, **p < 0.01, ***p < 0.001

## Slide 2
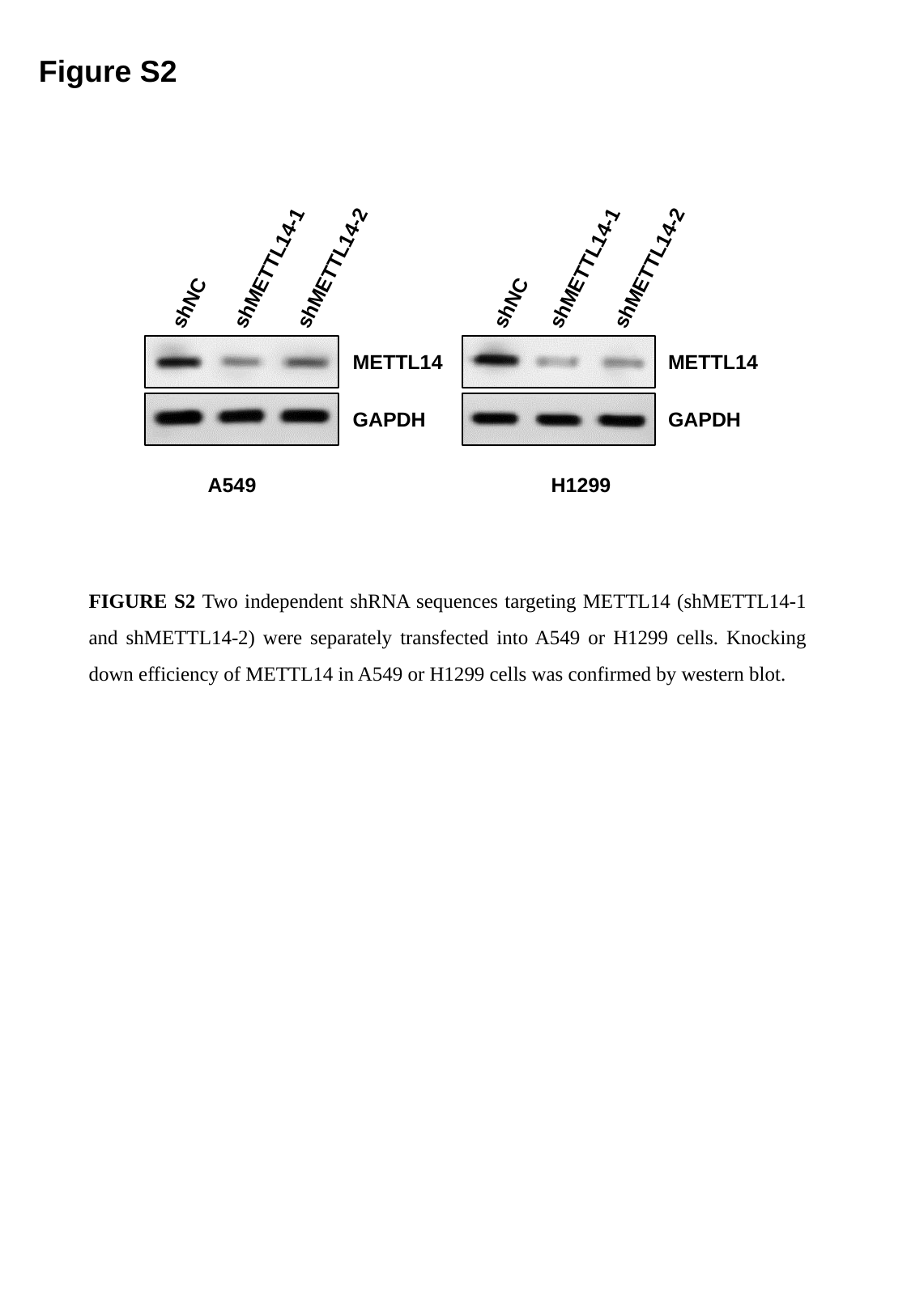

Figure S2
shMETTL14-1
shMETTL14-2
shMETTL14-1
shMETTL14-2
shNC
shNC
METTL14
METTL14
GAPDH
GAPDH
A549
H1299
FIGURE S2 Two independent shRNA sequences targeting METTL14 (shMETTL14-1 and shMETTL14-2) were separately transfected into A549 or H1299 cells. Knocking down efficiency of METTL14 in A549 or H1299 cells was confirmed by western blot.

## Slide 3
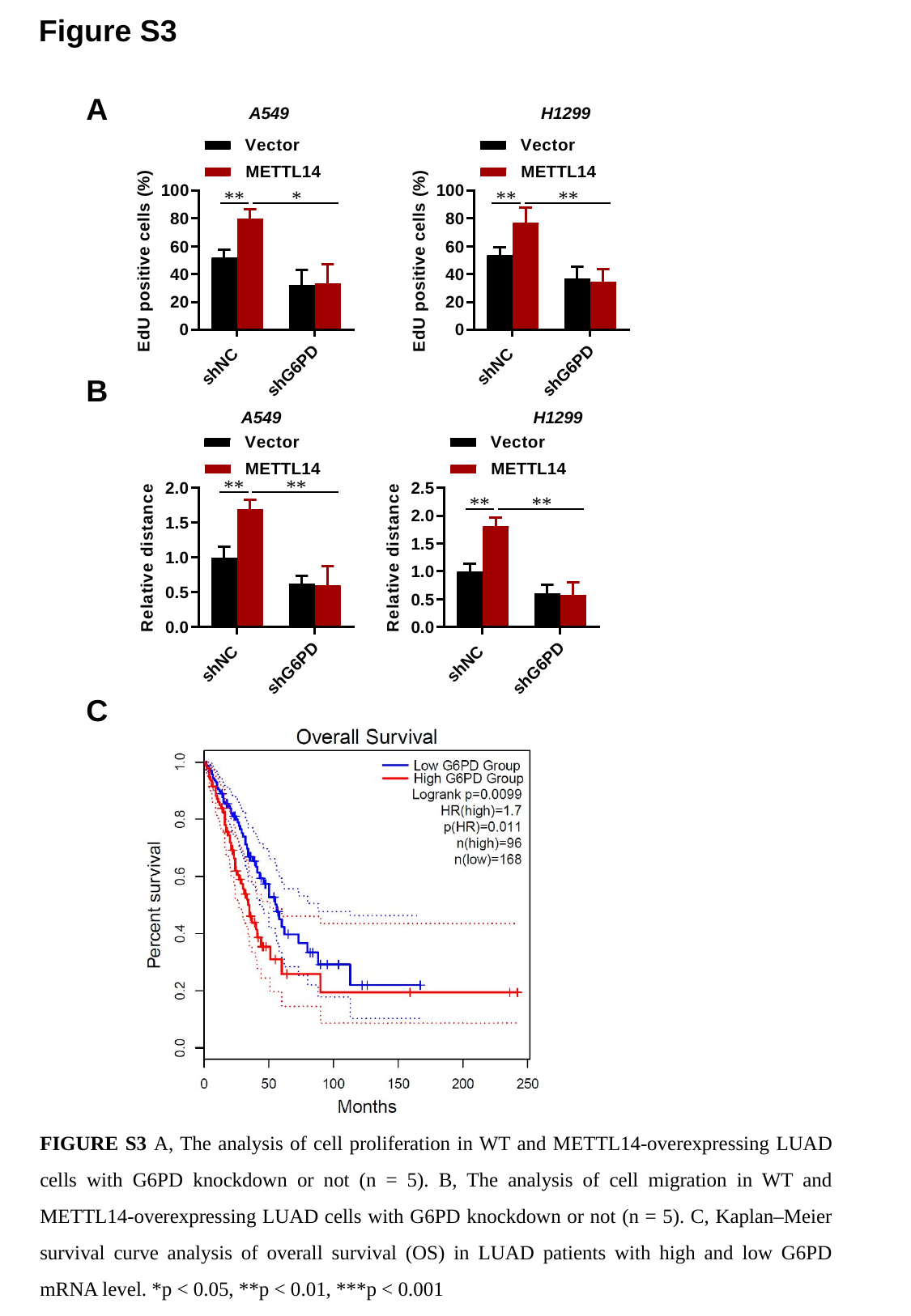

Figure S3
A
A549
H1299
**
*
**
**
B
A549
H1299
**
**
**
**
C
FIGURE S3 A, The analysis of cell proliferation in WT and METTL14-overexpressing LUAD cells with G6PD knockdown or not (n = 5). B, The analysis of cell migration in WT and METTL14-overexpressing LUAD cells with G6PD knockdown or not (n = 5). C, Kaplan–Meier survival curve analysis of overall survival (OS) in LUAD patients with high and low G6PD mRNA level. *p < 0.05, **p < 0.01, ***p < 0.001
