# Supplementary material for: METTL14-mediated m6A mRNA modification of G6PD promotes lung adenocarcinoma
Source: Cell Death Discov. 2024 Aug 13;10:361. doi: 10.1038/s41420-024-02133-w (PMC11322390; doi:10.1038/s41420-024-02133-w)
Supplement: Supplementary file 2 — Supplementary Table 1 [file 41420_2024_2133_MOESM2_ESM.pdf]

Supplementary Table 1：Primers used for qPCR assay.

Gene	Forward (5´-3´)	Reverse (5´-3´)
**G6PD**	CGAGGCCGTCACCAAGAAC	GTAGTGGTCGATGCGGTAGA
**IGF2BP1**	GCGGCCAGTTCTTGGTCAA	TTGGGCACCGAATGTTCAATC
**GAPDH**	GCACCGTCAAGGCTGAGAAC	GGATCTCGCTCCTGGAAGATG
**IGF2BP2**	AGTGGAATTGCATGGGAAAATCA	CAACGGCGGTTTCTGTGTC
**IGF2BP3**	TATATCGGAAACCTCAGCGAGA	GGACCGAGTGCTCAACTTCT
**METTL14**	GAGTGTGTTTACGAAAATGGGGT	CCGTCTGTGCTACGCTTCA
