# Supplementary material for: METTL14-mediated m6A mRNA modification of G6PD promotes lung adenocarcinoma
Source: Cell Death Discov. 2024 Aug 13;10:361. doi: 10.1038/s41420-024-02133-w (PMC11322390; doi:10.1038/s41420-024-02133-w)
Supplement: Supplementary file 3 — Full and uncropped western blots [file 41420_2024_2133_MOESM3_ESM.pptx]

## Slide 1
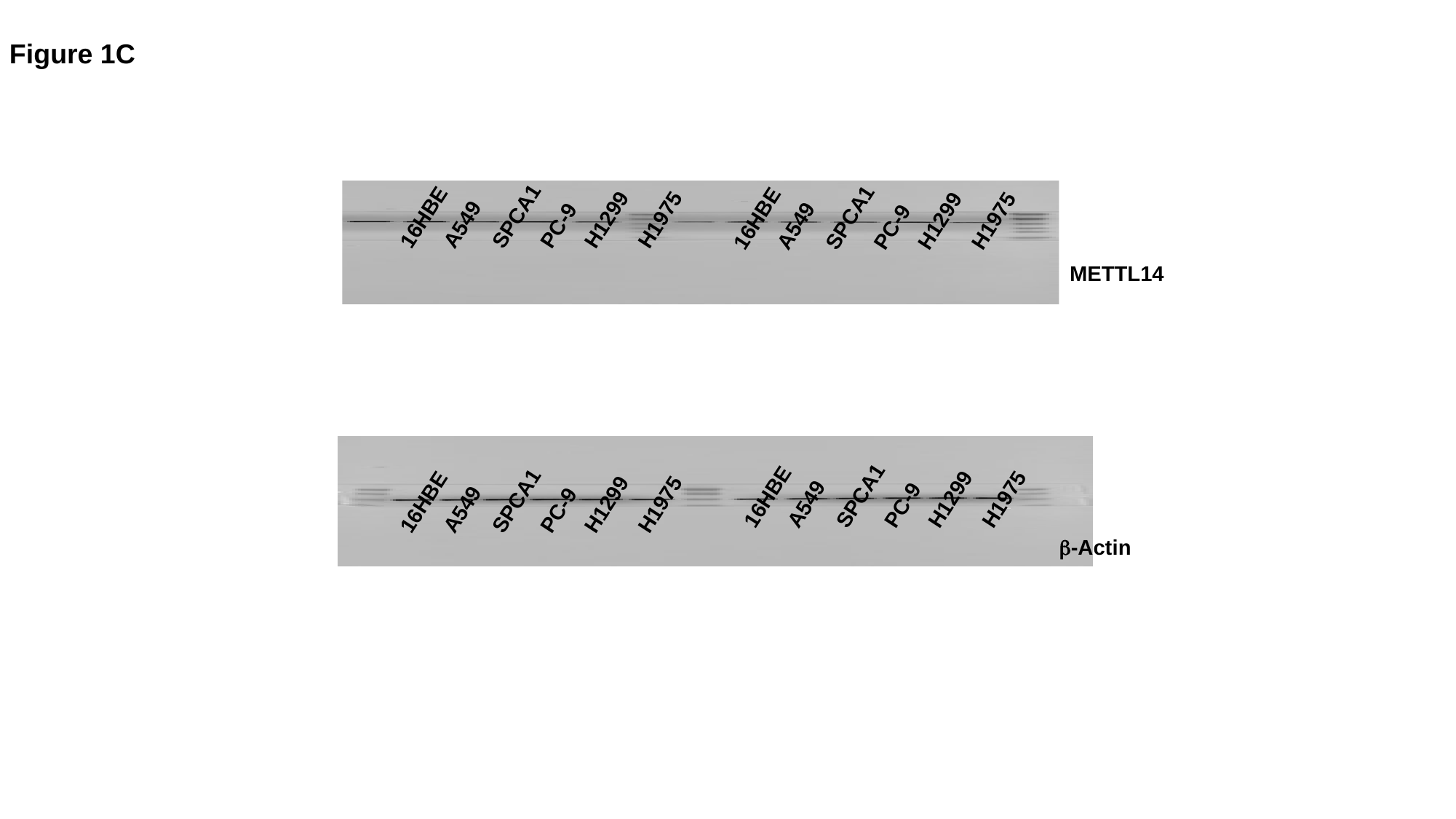

Figure 1C
16HBE
16HBE
A549
SPCA1
PC-9
H1299
H1975
A549
SPCA1
PC-9
H1299
H1975
METTL14
16HBE
A549
SPCA1
PC-9
H1299
H1975
16HBE
A549
SPCA1
PC-9
H1299
H1975
b-Actin

## Slide 2
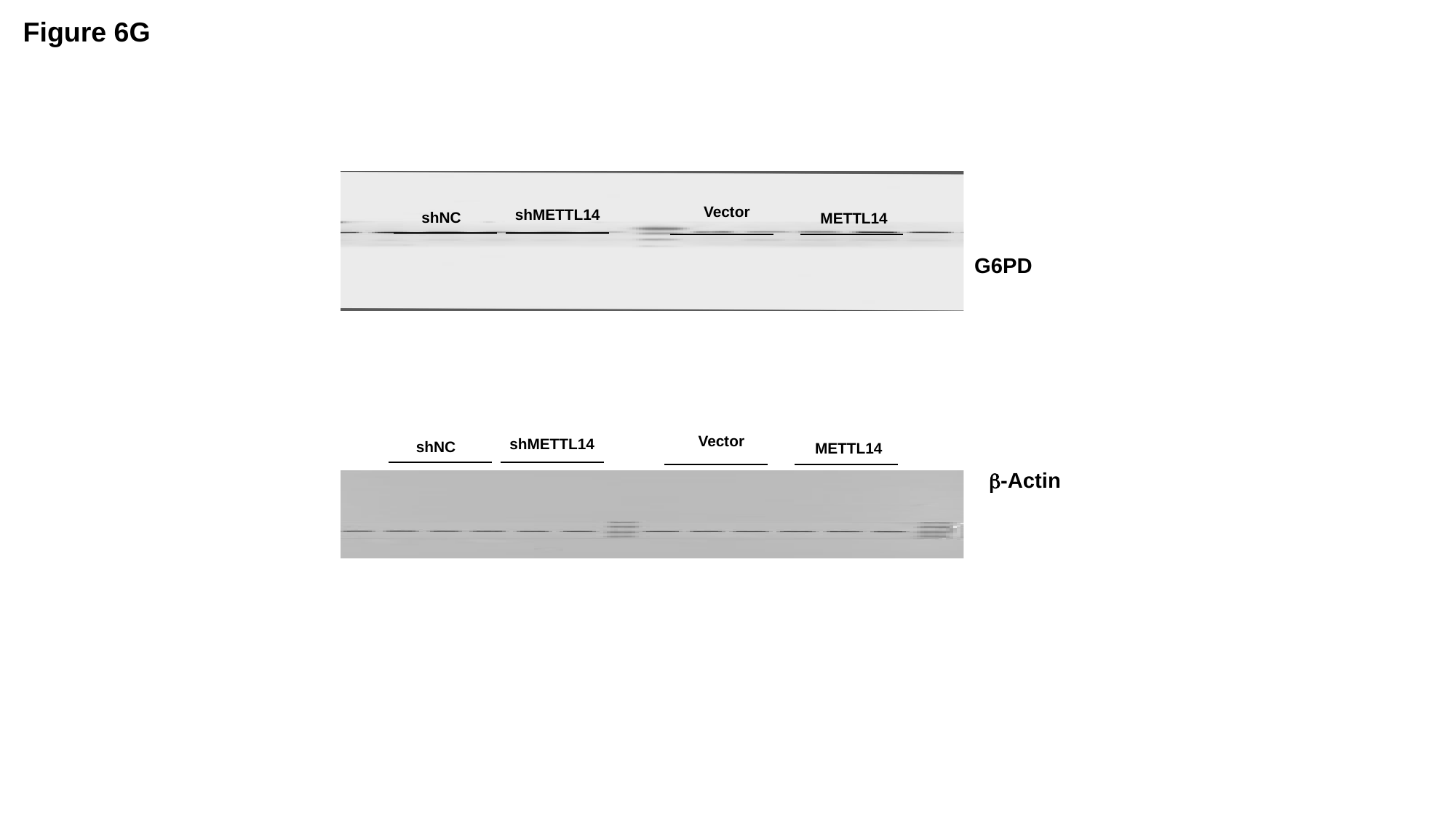

Figure 6G
Vector
shMETTL14
shNC
METTL14
G6PD
Vector
shMETTL14
shNC
METTL14
b-Actin

## Slide 3
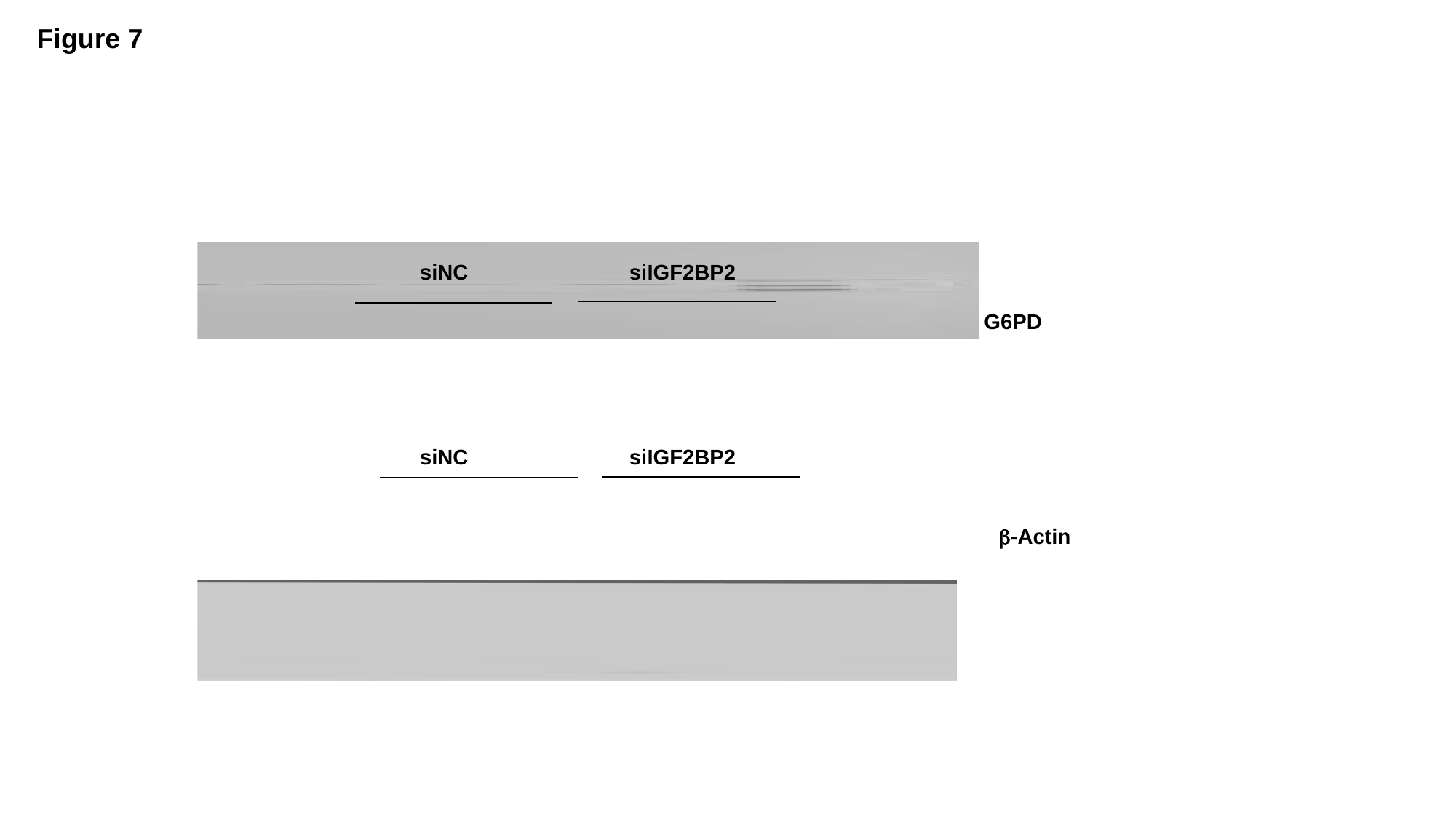

Figure 7
siNC
siIGF2BP2
G6PD
siNC
siIGF2BP2
b-Actin
